# An Alaska Native community’s views on genetic research, testing, and return of results: Results from a public deliberation

**DOI:** 10.1371/journal.pone.0229540

**Published:** 2020-03-16

**Authors:** Vanessa Y. Hiratsuka, Julie A. Beans, Jessica W. Blanchard, Justin Reedy, Erika Blacksher, Justin R. Lund, Paul G. Spicer

**Affiliations:** 1 Research Department, Southcentral Foundation, Anchorage, Alaska, United States of America; 2 Center for Applied Social Research, University of Oklahoma, Norman, Oklahoma, United States of America; 3 Department of Communication, University of Oklahoma, Norman, Oklahoma, United States of America; 4 Department of Bioethics and Humanities, University of Washington, Seattle, Washington, United States of America; 5 Department of Anthropology, University of Oklahoma, Norman, Oklahoma, United States of America; Chinese Academy of Medical Sciences and Peking Union Medical College, CHINA

## Abstract

As genetic testing technology advances, genetic testing will move into standard practice in the primary care setting. Genetic research, testing, and return of results are complex topics that require input from Alaska Native and American Indian (ANAI) communities as policies are developed for implementation. This study employed a day and half long public deliberation with ANAI primary care patients to elicit value-laden views of genetic research, testing, and return of results. Participants emphasized the need for a balance between the potential for genetics research, testing, and return of results to empower individuals and improve health with the potential to expose individuals and communities to privacy breaches, discrimination, and emotional harms. Public deliberation was well received by this group of participants and elicited rich discussion on the complex topic of genetic research, testing, and return of results.

## Introduction

Genetic research with Alaska Native and American Indian (ANAI) people has a complex history. Groups underrepresented in research have been subject to missteps and mistreatment by the research community. Transgressions in the ethical conduct of research have ranged from failures to be inclusive and transparent, share research results and other benefits, or respect group and individuals’ rights and confidentiality to engaging in research that stigmatizes ANAI people and violates cultural values [1–3]. Due to research harms that have occurred within genetic studies and health studies in general, many ANAI communities are understandably skeptical of participating in health research generally and genetic research specifically. As genetic research continues to advance, it has been suggested that there is an imperative to include ANAI communities to avoid widening health disparities [4, 5]. US Federal efforts, such as the *All of Us* Research Platform, have failed to consult appropriately with ANAI communities prior to targeted recruitment of ANAI people [6], despite ANAI tribal leaders’ clear concern [7].

Even with these abuses and continued missteps, some ANAI communities have recognized a benefit of participating in genetic research studies. Participation in genetic research, however, is prefaced with the expectation that research in ANAI communities requires community consultation [8–10]. Moreover, genetic research in ANAI communities must address areas of known disparities, clearly delineate potential health benefits and risks, and provide adequate protections against individual and group harm [11, 12]. ANAI communities expect transparent research practices that include tribal oversight and approval of the research, a robust informed consent process, and dissemination of study updates and findings throughout the research project [13–17]. Research partnerships that have taken the time to meet these expectations resulted in equitable partnerships with novel genetic findings [14, 16, 18–20].

Over the past three decades, US Federal and tribal policy makers and decision-makers in the health and bioethics sectors have increasingly sought public input into important policy questions, including questions raised by genetic research [21–23]. As genetic research advances and genetic testing yields medically actionable results, genetic testing will most likely increase in clinical practice, including tribal healthcare. Previous published qualitative work shows that ANAI community members are interested in receiving results from health research projects at both a community and individual level [17, 24, 25]. The return of genetic research results raises important value laden questions for ANAI communities to consider.

To gather public input on genetic-related issues, deliberative approaches have been used and are particularly well suited to discuss genetic research among specific groups, such as ANAI communities. This approach to stakeholder engagement convenes diverse members of the public to learn about, discuss and provide reasoned and informed input to decision-makers about important and often contentious social or policy issues [26, 27]. Because topics, like genetics, are complex, value-laden, and subject to debate, deliberation involves careful consideration of competing perspectives, underlying values, and tradeoffs [28]. Deliberative engagement complements other forms of gathering public input, such as surveys and focus groups, by seeking carefully considered (rather than top-of-mind) opinions that are civic-minded and oriented toward finding common ground [29–33].

This manuscript describes a two-day community deliberation that facilitated discussion among ANAI primary care patients about genetic research, testing, and return of results. The aims of this deliberation were to (1) test public deliberation among ANAI community members, (2) engage in meaningful discussion on the complex topic of genetic research, testing, and return of results with ANAI community members, and (3) to elicit ANAI community member perspectives on genetic research, testing, and return of results in their community. These data were summarized and disseminated with the intention of informing the ANAI community and ANAI leadership for future healthcare planning.

## Materials and methods

**Fig 1:**
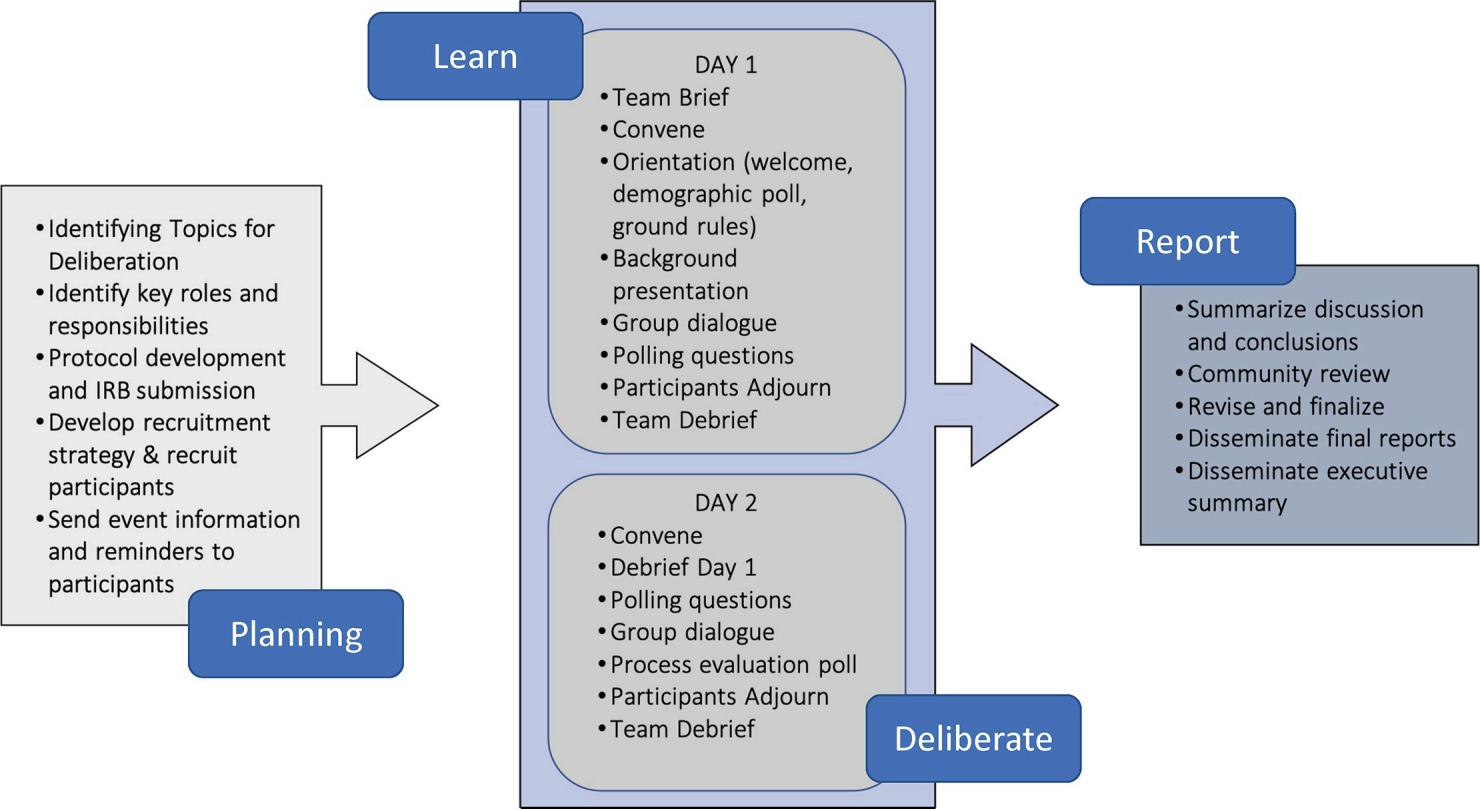
Public Deliberation: Process and core elements. Figure adapted from Carman et al. (2015). [34].

This project was reviewed and approved by all appropriate Institutional Review Boards and SCF research review committees and Board of Directors. Additionally, this manuscript received tribal approval from SCF research review committees and Board of Directors prior to journal submission [9]. The deliberative community engagement methodology allowed for ANAI community members to have a nuanced discussion on genetic research, testing, and return of results in this tribal health care system. The study design intentionally encouraged engagement throughout the deliberation by integrating large and small group discussion, surveys, and interactive exercises to ensure individuals with varying levels of comfort had the opportunity to participate (Fig 1).

### Setting

Southcentral Foundation (SCF), a non-profit tribal healthcare organization, provides a wide range of health and human services to more than 65,000 ANAI people in the Indian Health Service Anchorage Service Unit, a population that includes both urban and remote rural ANAI villages. The setting for this study provided a unique research opportunity as this study was conducted within a closed healthcare system in which pharmacogenetic research has been conducted previously [13, 14, 16–18, 20, 35]. Customer-owner is SCF’s term for ANAI community members who are self-determined customers of their health care system [36]. At SCF customer-owners are viewed as experts in determining how health care services are to be delivered and prioritized. Public deliberation aligns with SCF’s relationship-based approach to healthcare and emphasis on the need for community-driven healthcare priorities.

To drive transparent research practices and overcome power differentials with researchers outside of the ANAI community in the south central region of Alaska, SCF created a Research Department in 2007 to conduct research for ANAI people by ANAI people [9]. Two ANAI researchers (VYH and JAB) with the SCF Research Department led the SCF deliberation project in partnership with the University of Oklahoma [37]. To help familiarize SCF leadership with public deliberation as a research method, the research team developed and provided an overview of deliberation for SCF leadership to consider. Once SCF leadership approved deliberation as a research method they then decided on the deliberation topic: genetic research, testing, and return of results.

The deliberation occurred in January 2019 over a two-day period on the Alaska Native Medical Center campus in Anchorage, Alaska. The deliberation was facilitated by two study team members, a bioethicist and deliberation scholar (EB) and an ANAI mixed methods researcher (JAB). Three additional study staff (JWB, JR, JRL) were also present throughout the event to observe, take notes, and assist as needed throughout the deliberation, with additional staff on hand to assist with registration, food preparation, and facility management.

### Sample

A convenience sampling frame was used to recruit 30 ANAI adults, who received services at SCF in the last two years and were able to participate in the day and a half deliberation. Recruitment took place through in-person recruitment tables in the lobby of the SCF Anchorage Native Primary Care Center. During recruitment, research team members explained the purpose of the deliberation event as well as other specifics of participation, such as length of deliberation and voluntary participation.

Potential participants were provided information about the deliberation process and, interested individuals registered for the deliberation event by providing their name and contact information. Registered individuals were given contact information for research team members if they had questions prior to the deliberation event. We used the contact information to send interested individuals a reminder of the deliberation dates and time as well as an agenda (Supporting information S1 Appendix).

### Deliberation event

This community engagement was designed in the form of a public deliberation to increase participants’ understanding of genetic research and return of results in relation to each individual, family, and community; and to capture community members’ comments, concerns, and possible utility of genetic research and return of results (Fig 1). The goal of this deliberation was to understand ANAI community perspectives and recommendations about genetic research, testing and return of results and determine whether genetic research, testing and return of results may have value to participants’ families, their community, or themselves. We aimed to hear a full range of views, and the values and reasons behind them. The event was designed to allow participants an opportunity to interact with one another in different groupings over the course of the day and a half even and sought to provide a comfortable environment for all participants to share personal views.

All participants registered with study staff the first day of the deliberation event. During registration each participant was provided a packet that included an informed consent form and deliberation materials. Each participant reviewed the informed consent form, then a study staff member reviewed the informed consent form with the participant and allowed time to respond to questions. Each participant was then asked to provide verbal informed consent including consent for audio recording. All participants provided verbal informed consent, and once enrolled were asked to complete a demographic form.

The deliberation began with an ANAI presenter (VYH) providing participants with information about genetics and genomics research and testing and the conduct of research at SCF, and opportunity to ask questions (Supporting information S2 Appendix). During the deliberation participants discussed three issues within genetic research, testing and return of results at SCF: (1) potential risks; (2) potential benefits; and (3) questions about governing policies.

### Deliberative exercises

To encourage discussion, we used hypothetical scenarios (Supporting information S4 Appendix). All scenarios were drafted by an AI doctoral candidate at the University of Oklahoma (JRL) in consultation with ANAI community-based and placed researchers at SCF (VYH and JAB). An interdisciplinary review was provided by the entire study team prior to scenario finalization.

Participants were provided scenarios on genetic research, testing and the return of results, then given time for small group discussion. The scenarios served two purposes: 1) to break the participants into small groups to provide individuals who may feel uncomfortable speaking in the large group setting an opportunity to voice their opinions; and 2) to present a nonpartisan perspective, offering participants both potential pros and cons, and to inspire further discussion. The scenarios were designed to successively build up to the complex topic of return of results while challenging the deliberants to consider a small spectrum of issues and how they might feel about them.

Over the course of the event, six scenarios were presented to the participants in small groups detailing cases that explored genetic research, testing and return of results, including direct-to-consumer genetics tests. Each group thoroughly discussed a scenario and its implications. The small groups then reported back their case and their discussion to the larger group for further comment from the plenary group. On Day One, participants were presented two cases that generally described a situation to consider about genetics and genetics research (Supporting information S3 Appendix). These scenarios encouraged participants to think about the risks and benefits of genetics and genetics research relation to the individual, family and community. Two additional breakout sessions were guided by scenarios on Day Two that presented the participants with situations that challenged them to consider the situation where one could receive confusing, distressing, or problematic results, both clinically as well as through direct-to-consumer testing (Supporting information S4 Appendix).

During plenary group scenario discussion, issues of importance expressed by participants were documented on flip charts. Participants were then asked to discuss, prioritize, and select their top two considerations. This process of documenting participants’ concerns, followed by the prioritization and selection of only two concerns, encouraged participants to reflect on and exchange the available choices to precisely identify the issues and values that resonated for them at that time. Identifying resonant issues offered concrete points for ongoing deliberation and created space for participants to shape the discussion based on their own priorities and emergent themes.

Participants also completed an 18-item opinion poll three times throughout the deliberation (Supporting information S1 and S3 Appendices). The polling questions were designed to stimulate specific situations participants might find themselves in. For example, some polling questions asked the participants their opinions on genetics research while others inquired about genetic tests and results. Additionally, an effort was made to use site-specific terminology and familiar medical encounters and services.

### Post deliberation survey

At the conclusion of the event, participants completed a 16-item survey on event satisfaction and perceptions of the discussion. Each participant received $300 in gift cards for their participation.

### Deliberative quality assessment

**Fig 2:**
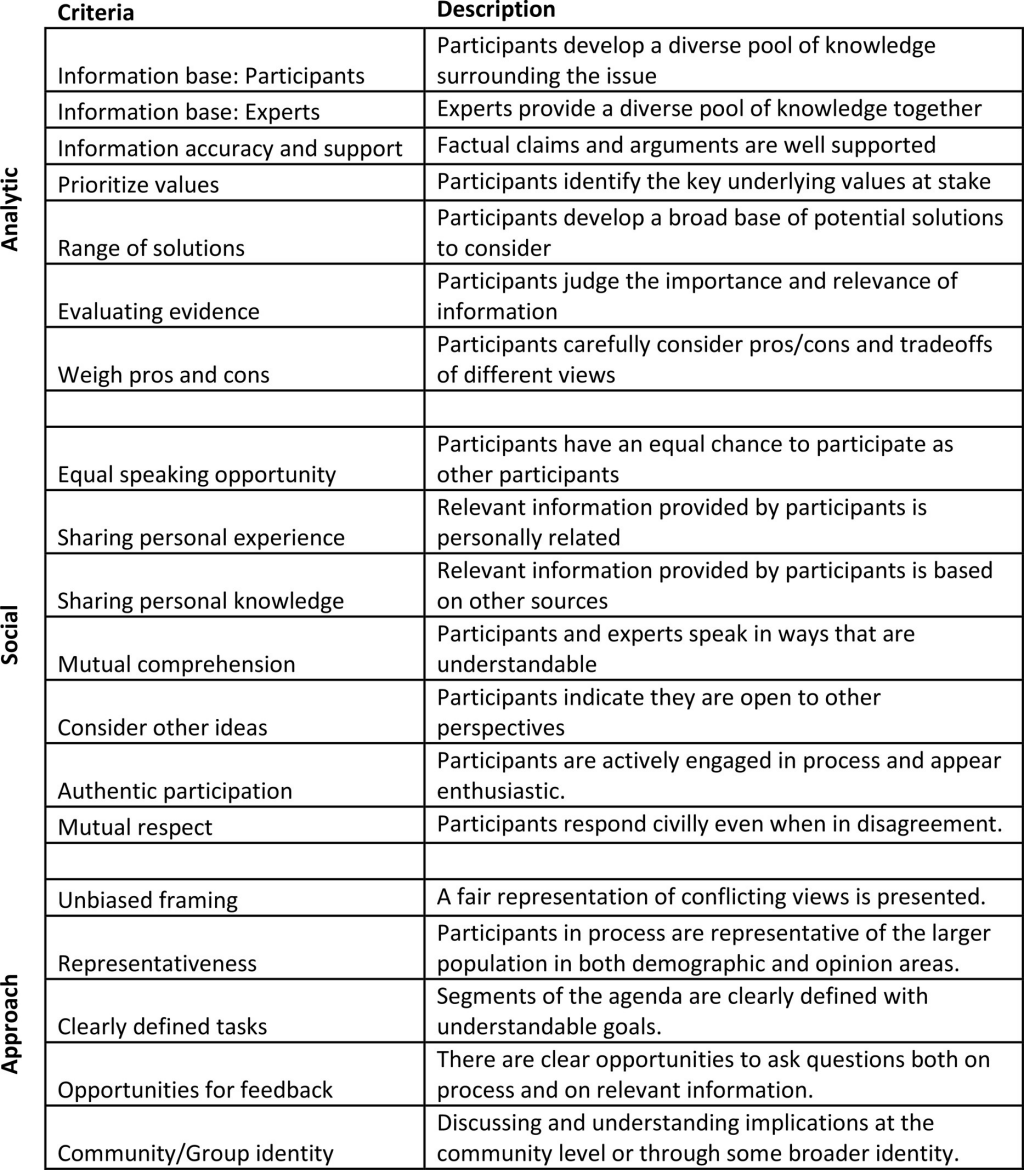
Deliberation assessment criteria.

To evaluate the quality of the deliberation, an observer (JR) took field notes throughout the deliberation and adapted previously validated forms of deliberative evaluation criteria for this context [38]. Following each day of the deliberation, a coding sheet was completed to assess the design and intent of the deliberative process itself, and the actual deliberative experiences of the participants at the event (Fig 2). This evaluation measured event planning success and democratic process execution that led to thoughtful, robust deliberation among participants. These criteria are separated into three main categories: 1) the analytic components of deliberation, which focused on the information quality in and analytic rigor of the process; 2) the social components of deliberation, which focused on the nature of the social and group interactions within the deliberation; and 3) the approach of the event, which focused on several other important components of a democratic process of stakeholder engagement, and is based on other work evaluating such events [39–41]. The deliberation was assigned a score on each of the criteria ranging from zero to five, with zero representing never occurring and five representing always occurring (and N/A indicating criteria that were not applicable in a particular segment), for each of three major segments of the event—the evening of Day One, the morning of Day Two, and the afternoon of Day Two.

### Summary report to participants

In the week following the deliberation, we developed a summary report to describe the main concerns and suggestions regarding the return of results from genetic tests. A draft version of the summary report was sent via US mail to all participants for review and feedback (a stamped return envelope was provided) to ensure the community views were described accurately and appropriately. Each participant who returned feedback on the summary report was compensated for their time with a $25 gift card.

### Analysis

#### Qualitative analysis

The qualitative analysis for this paper was limited to a cross-comparison content review of detailed notes taken by multiple observers (JR, JWB, JRL) during the large and small group discussions to summarize general views about genetic research, testing, and return of results in a tribal health setting. This content review was coupled with a preliminary thematic analysis of the notes to further contextualize general views that emerged during the deliberation. The deliberation recordings were transcribed verbatim and used to supplement the notes as needed, though a detailed thematic and discourse analysis of the deliberation will be the focus of subsequent papers [23, 28, 40, 41].

Given the uncertainty about how the deliberative process would be received by participants, we maintained rather loose expectations about the specific nature of the qualitative data that might emerge out of this deliberation. The deliberative setting generates data that is inherently *dialogic*, meaning that the data embodies the participants’ “collective efforts to explore meanings and interpretations of the information presented for deliberation” (p.2) [42]. Deliberation promotes a space for dialogue among participants where they find ways, as a collective and as individuals, “to express, examine, or extend collective understandings of complex concepts” (p.2) [42]. The resulting data, therefore, encompasses both the stated perspectives of the participants but also provides a window into the process whereby meaning emerges through the exchange of these perspectives.

#### Quantitative analysis

Brief descriptive summary statistics were calculated for all survey and poll responses. Individual poll responses were assessed with Likert scale changes noted over the course of deliberation. Particular attention was paid to areas where views changed from agree, disagree or unsure [33, 38, 40].

## Results

Nineteen customer-owners (11 female, 8 male) participated in both days of the deliberation. Participants ranged in age from 22–63 years and reported varied levels of education from high school to post-baccalaureate. Day One of the deliberation, participants spent time discussing the presentation points and understanding the role of research versus clinical care. Participants asked questions to further understand what the boundaries of genetic research might be. For example, one participant asked if genetic results from research could be used in clinical care. Participants also sought clarification around the timeline of research. After taking the first set of polling questions, some participants made comments saying the questions, “…had me thinking.” Another participant shared, “Most of my unsures were just like–well, I have no clue what the implications of genetic research are.” Participants proceeded with an in-depth discussion about the polling question constructs, and this process of participants commenting on the various data collection methods continued through the completion of the event.

All participants returned on Day Two and some shared that they thought through the discussion from Day One. The first scenario discussion on Day Two, participants had fewer questions on what genetics was and began to put forward their views on what aspects were important. A turning point in the conversation, however, happened during the plenary group discussion where salient issues for the group were documented on flip charts. As participants saw their concerns written out, many began to pinpoint group concerns and think about the larger picture of genetic research. For instance, one participant described bioethical dilemmas regarding eugenics and the slippery slope of gene editing in their statement:

*“So…I’m not sure how far along this would go down the line*. *It could be many years, it could be decades, it could be centuries, but where do we decide where the ideal health is? We can fix one problem, we can fix certain disease that will really affect people, but could we really decide where it stops and where the most healthy person is? You know, there’s that kind of idea like perfect person, designer babies*. *How far should we go?”*

Another participant reflected on potential behavioral support needs when receiving test results as they stated:

*“So we don't know what's gonna happen with our testing as the test, but at some point if it's decided that somebody should receive the results of the testing are they ready for those results? Are they gonna be suicidal or*, *you know, are they gonna find out that, oh, you know*, *like the baby thing–oh, you know, for some crazy reason–this is hypothetical and not with research–my testing contributed to abortion and I didn't know that was gonna happen.”*

Participants identified a wide range of issues and questions about the potential benefits, risks, and policy implications related to genetic research, testing, and the return of results. The potential benefits and risks of genetics research surfaced most powerfully (Table 1).

**Table 1:** Benefits and risks pertaining to genetic research, testing, and return of results discussed at deliberation event.

Benefits	Risks
Individual empowerment (‘knowledge is power’, “My body, my choice”) • Personally useful (e.g., can know what to expect and can take steps to improve health) • Insight into identity and family history, reconnecting families • DTC* tests may give individuals access to tests that healthcare providers may choose to deny Disease prevention and improved public health • Individual—Early detection, improved preventive care, better support systems (e.g., health education and counseling) for individuals • Collective—Public health knowledge (more knowledge, more initiatives) • Raised awareness and empowerment for community and tribal leadership • Improved understanding of why some communities are sicker than others • Improved understanding of role of environmental and social conditions • Emphasis on healthy ANAI traditional diets and activities (e.g., fishing, hunting	Privacy Breaches and Misuse of Results • Unintentional or intentional breaches that reveal personal identities and genetic information • Challenges of maintaining individual privacy in a health care system that include close social and family relationships (e.g., ““we all know people here at the hospital”) • Discrimination in employment or healthcare insurance on basis of one’s genetic results • Who controls access to information, for what purposes would it be shared, and whose property are the results? And, who decides these matters? Return of result challenges • Mistakes/errors (e.g., validity of results, wrong results) • Interpretation of results (e.g., uncertainty, what does it mean for my health?) • Effective communication (e.g., languages other than English, culturally appropriate, plain language, understood across generations) • Poor provider communication (e.g., causing confusion or misunderstanding) • Emotional impacts (e.g., worry, fear, distress, substance abuse, suicide)

*DTC–Direct to Consumer

Specifically, participants described the potential for genetics research and testing to empower and improve health at the individual and population level (Table 1). Participants also expressed hesitation regarding genetic research and testing and described genetic research and testing as a possible opportunity to expose individuals and communities to privacy breaches of personal information, discrimination, and emotional harms (Table 1). The broad discussion makes it difficult to characterize participants’ perspectives about genetics as either positive or negative; indeed, participants carefully weighed the positive and negative impacts of genetic research so that statements about the potential benefits of genetics were frequently offset by considerations of the risks (and vice versa). Table 1 highlights more salient issues across the group, suggesting that participants prioritize both benefits and risks in their selections.

The deliberants’ discussion underscored the many ways that participants evaluated tradeoffs with genetic research and testing. In summary, genetic research is capable of promoting family connections, but may also expose paternity information in harmful ways. Some participants saw direct to consumer genetic testing as an empowering means to participate in their own preventative health care, but also questioned the unregulated nature and credibility of these tests. Many discussed the importance of engaging family members in discussions about genetics as part of their overall approach to care, but also expressed concerns that including families in personal health care decisions could challenge their individual autonomy and privacy.

Participants also discussed the impacts of genetic research, testing and return of results in ways that highlighted important distinctions related to individual versus collective concerns. Many of the potential benefits of genetic research involved the promotion of individual autonomy, privacy and empowerment to make informed decisions. Individual autonomy was a central theme throughout the discussions, and participants expressed concern that genetic research that promises collective benefits or value might also diminish an individual’s autonomy. It is noteworthy that much of the discussion highlighting the *collective* impacts of genetic testing had less to do with the potential for collective benefits and more to do with collective harms, including shared histories of trauma, communal harm associated with loss of traditional dietary practices and damage to shared environments. These stories of collective harm seemed to resonate with others in the group, serving as a basis for understanding the potential risks involved in genetic research and the potential for genetic research to perpetuate collective mistrust and violate communal values. Establishing a clear distinction between the “cultural roots” and values of AN communities and those who may conduct genetic research, a male participant spoke to the importance of awareness and mindfulness when assessing the future of genetic research. He explained that helping others is a priority “in our Alaska Native culture but that’s not everyone’s morals. Pay attention to the morals of the research and make sure it is protected.”

Beyond considerations about the potential benefits and risks of genetic research and testing, participants engaged deeply with issues about the questions that would need to be addressed if genetic testing and return of results became routinely integrated into healthcare at SCF. The theme of individual and collective concerns emerged again in these discussions and was embedded in the conversations about policies governing genetic research in the local healthcare system. There were clear expressions of interest in the potential for genetic testing and return of results to empower individuals in making their own health care decisions, with a reoccurring reminder that such decisions should be made against the backdrop of a long history of collective harms and injustices. For some participants these concerns were part of broader mistrust of genetic research and testing, the role and motives of big corporations, especially pharmaceutical companies, conflicts of interest, and non-tribal systems and researchers.

### Polling

The polling results show a change in participants’ views towards various aspects of genetic research and genetic tests within SCF. For some questions, participants went from being unsure about the question in the first poll to taking an agree or disagree stance in the last poll. For instance, nearly half of participants (9 out of 19) were unsure of their views at first on questions about allocating funds for genetic tests or relevant support (e.g. genetic counseling) even if that meant fewer funds for other health programs; by the end of the deliberation most participants solidified their views on this, with most agreeing that SCF should fund such genetic tests (11 out of 19) and support for those who take tests (14 out of 19). Similarly, some participants were unsure about a statement that is important for people to know the results of genetic tests even if they do not know what the results mean; by the end of the deliberation all participants had taken a stance, with 15 out of 19 people agreeing that it is important for people to know the results of genetic tests even if they do not know what the results mean. The level of discussion that took place throughout the deliberation highlighted in the qualitative results are reflective of participants’ changes in perspective on genetics research, test and return of results.

### Post deliberation survey

Overall, participants understood the purpose of the event, felt that participating in the deliberation was a good use of their time and that their recommendations will influence SCF approach to genomic medicine going forward. In agreement with the qualitative data and the polling questions, the majority of participants (13 of 19) agreed that the discussion led them to change some of their opinions on genetic research and testing and 17 of 19 participants thought the group discussion affected their opinions of genetic research and testing. The level of engagement by the group was demonstrated by 18 of 19 participants agreeing they were interested during group discussion.

### Deliberative quality assessment

**Fig 3:**
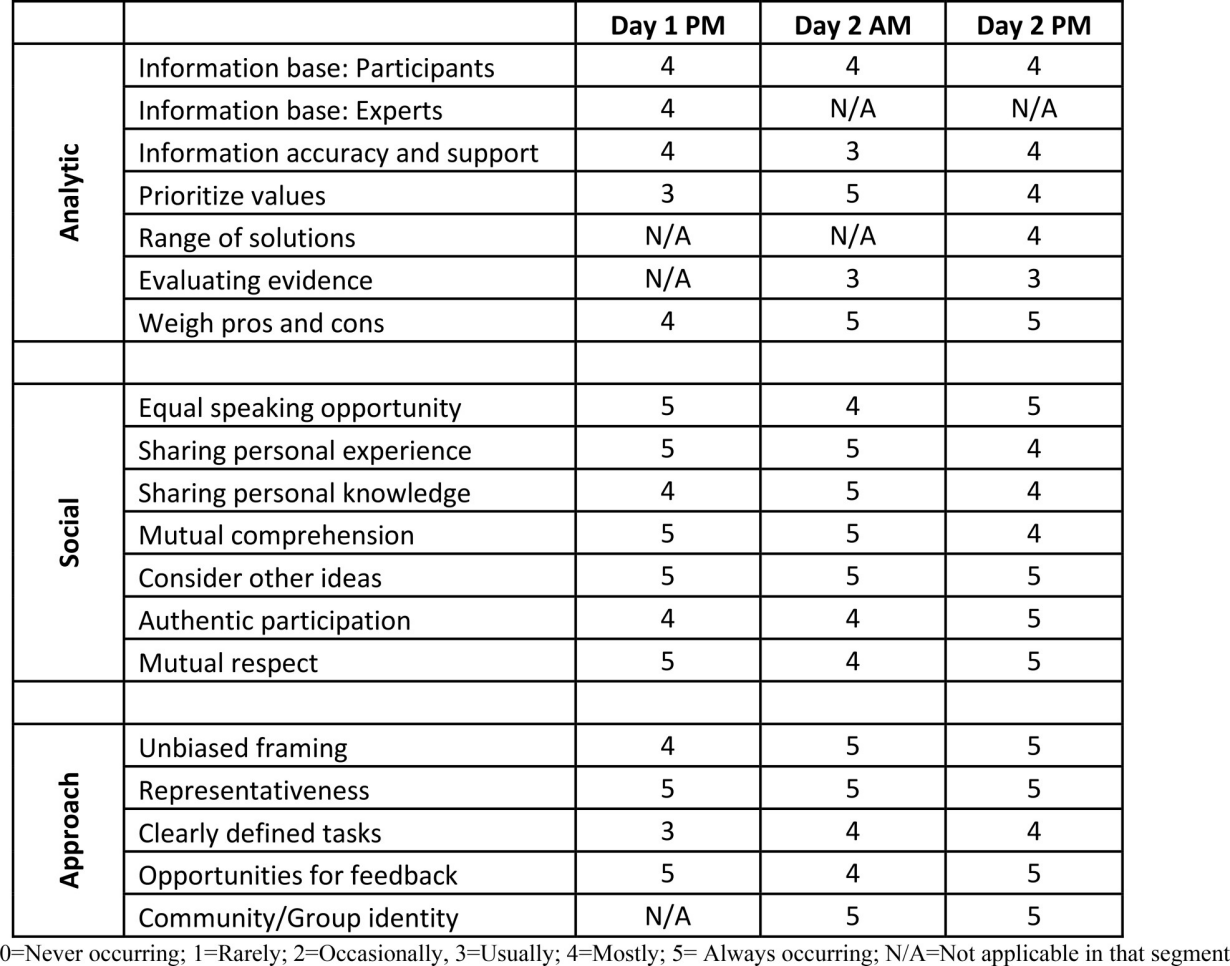
Assessment of deliberative event.

The deliberative quality of the event was rated very well on nearly all the assessment criteria, with most of the key markers of deliberation happening mostly or always during each of the major segments of the deliberation (Fig 3). The deliberation was rated especially high on the social components of deliberation, such as providing opportunities for everyone to speak, exhibiting mutual respect, participating authentically, and comprehending and considering what others had to say. The deliberation also did well in most of the analytic components of deliberation, such as the quality of information and knowledge developed and shared by participants, weighing of pros and cons of different options, and prioritizing the key values that are at stake around the issues of genetic tests and return of results.

Some components were less well represented in the process but were still present in most of the discussion segments, such as the critical evaluation of evidence and providing support for factual claims and arguments. A few criteria were less applicable to the entirety of the process, but were well represented at some point during the day and a half event, such as the information base provided by an expert, which occurred in the evening on Day One, or a discussion of several potential policy choices or solutions, which was more of a focus in the afternoon of Day Two. The deliberation also fared well in the several criteria for the approach guiding the event, such as giving an unbiased framing to the topic of discussion, ensuring the group is fairly representative of the larger population (in this case, the ANAI population in Alaska served by SCF), giving participants opportunities for questions and feedback on both the process and topic, and in helping participants understand the issue in terms of the broader community concerns.

Most participants remained actively engaged in the deliberation until the end, with all individuals participating at some point during the event. Participants remained positive about the process until the end, and many individuals offered concluding remarks that expressed satisfaction with the event. These remarks included comments about the benefit of learning what others think, an appreciation of the process, the desire to pursue more information about the topics discussed, and an appreciation of the opportunity to participate. For example, when one of the moderators asked how the forum went for them, one participant remarked on how much they had learned about the topic:

“*Before coming to this research study*, *I did not now what genomics was or why it’s important to think about*, *and now that we’re done*, *it’s good to know that we can find certain diseases by the markers*, *even if we do have them it doesn’t necessarily mean that wee will get them*. *It just means that we’re at more high risk…”*

Another participant brought up their appreciation of learning more about their fellow participants’ view on the topic:

“*I like coming to research groups and I would like to be part of more*. *It’s very eye opening*. *I knew just a little bit about genomics but seeing everybody on a different level of where they value verses where I was*, *you know–like not everybody is going to be on my same page and that’s great because then it gives you more insight and more information*. *It’s great*.*"*

It was evident over the course of two days that participants forged new interpersonal relationships with each other, outwardly expressing support for one another and offering concluding expressions of gratitude for the opportunity to experience different tribal cultures, hear a range of perspectives that they noted “were all valid,” and feeling “lucky” to have been able to meet “awesome people.” Participants expressed interest in additional opportunities to participate in research with SCF.

### Summary report feedback

Nine of the nineteen participants returned feedback on the summary report and the summary report was revised based on feedback. All participants who returned feedback on the summary report agreed with the summary, however, the presentation of the summary was described as too complex. In response, we moved text of the findings into a table, added in bullet points, and more white space to the document to improve document readability. A final copy of the summary report (Supporting information S5 Appendix) was sent to all participants who indicated they would like a copy. A final summary report was also submitted to SCF Research Oversight Committee, Executive Committee, and Board of Directors for consideration in the development of genetic policy at SCF.

## Discussion

This public deliberation study sought views from ANAI patients about genetic research, testing, and return of results in an ANAI serving healthcare setting. To our knowledge, this is the first study to describe and report on the use of public deliberation as a research method in a tribal setting. We found community members to be fully engaged throughout the deliberation, and they reported appreciating the opportunity to learn and discuss the complex topic of genetic research, testing, and return of results despite having no prior knowledge on the topic. These findings showcase the acceptability of deliberation as a form of community engagement in genetic research when conducted in a culturally congruent manner. Our findings demonstrate not only the extent of the complexity involved with genetic research, testing, and return of results in tribal settings, but also community members’ ability to discuss and weigh the complex considerations involved with genetic research, testing, and return of results in a tribal setting while having no formal training in the topics of the ethics or science of genetic research and testing.

Participants dialogued on the concepts of community self-determination and individual self-determination as they discussed collective harms with less dialogue devoted to collective benefits. Rather, benefits highlighted through discussion placed emphasis the individual. These data introduce an interesting and challenging juxtaposition between individual benefit and group harm. Given that genetic medicine often focuses on individualized or personalized medicine, the emphasis on personal benefit rather than group benefit is not surprising. Realizing and communicating the collective benefits, along with individual benefit and group and individual harms, of genetic research, testing and return of results to ANAI communities may aid to facilitate conversations to include ANAI groups in genetic research and testing.

ANAI community members expect to be included throughout the entire research process, and do not find it acceptable to invite community participation only during research recruitment and dissemination of results [12, 25, 43]. Public deliberation and other community engagement activities held prior to the institution of new medical technologies, such as genetic research, testing and return of results in the primary care setting, provides community members an opportunity to take part and have influence on the decisions being made about the development of implementation policies and protocols relating to new technologies affecting their communities. Communicating these practices of transparency helps to solidify trust with the ANAI community by showing how researchers are working to include the ANAI community in the research process as early as possible. The views shared in the community deliberation echo participant feedback from ANAI community focus groups from over a decade ago [12, 43], and other qualitative inquiry more recently conducted at SCF [13, 15, 17, 25].

In addition, in-depth conversations that take place during the deliberation deepens community awareness of pressing issues within research involving the community. As noted in the results the conversation throughout this deliberation progressed from the participants being unsure of what genetics in their community meant to establishing succinct stances on what should be considered as genetic research return of results policies are developed. From conversation throughout the deliberation and in the literature, we know that participants continue to discuss these topics beyond the deliberative event, and in doing so, they spread awareness of these important topics [44, 45]. Building a greater knowledge base and consciousness of complex topics such as genetic research, testing and return of results is instrumental in the development of equitable policies and procedures. These findings provide reasoned and informed community input to tribal decision-makers about this important and complex issue to inform genetic policy development.

There are several limitations to this study. This study may not be generalizable as this was a convenience sample of ANAI primary care patients and participants were limited to those who could be present at the event in Anchorage for two days. Recruitment took place in the health care setting, and participants may have a closer relationship with the SCF primary care center than the majority of ANAI people who receive health care services from SCF which could bias the views shared in this study. Study participants were also mostly women, which may not adequately represent male views in this group. The quality assessment was conducted by a single observer which may introduce bias to those findings.

To translate the perspectives from the ANAI community deliberants into healthcare system policy, solicitation of additional input is required from SCF stakeholders including tribal leaders and clinical providers. The ANAI community members within our deliberation had complex considerations and expectations of involvement in genetic research, testing, and return of results and their perspectives should be considered when developing policy on these matters as their health care will be directly impacted.

## Supporting information

S1 Appendix Deliberation agenda. (PDF)

S2 Appendix Presentation discussion points. (PDF)

S3 Appendix Opinion poll. (PDF)

S4 Appendix Scenarios. (PDF)

S5 Appendix Final summary report. (PDF)
