# Supplementary material for: An Alaska Native community’s views on genetic research, testing, and return of results: Results from a public deliberation
Source: PLoS One. 2020 Mar 16;15(3):e0229540. doi: 10.1371/journal.pone.0229540 (PMC7075569; doi:10.1371/journal.pone.0229540)
Supplement: S1 Appendix — (PDF) [file pone.0229540.s001.pdf]

S1 Appendix. Deliberation agenda

Agenda

Friday, Jan. 25, 2019

4:30 p.m.	Registration and Informed Consent
5 p.m.	Dinner and Welcome
6 p.m.	Break
6:15 p.m.	Presentation – Genetics/Genomics
7:15 p.m.	Group discussion
8:30 p.m.	Adjourn for Evening

Saturday, Jan. 26, 2019

8:30 a.m.	Breakfast and Welcome
9 a.m.	Debrief from Day 1
9:30 a.m.	Story – Genomic Return of Results
10 a.m.	Break
10:15 a.m.	Group Discussion
10:45 a.m.	Identification of Important Issues
11 a.m.	Group discussions
12 p.m.	Lunch
1 p.m.	Story – Commercial Genetics Test Results
1:30 p.m.	Group discussions
2:10 p.m.	Break
2:30 p.m.	Identification of Important Issues
3:30 p.m.	Wrap up
4:30 p.m.	Adjourn
