# Supplementary material for: An Alaska Native community’s views on genetic research, testing, and return of results: Results from a public deliberation
Source: PLoS One. 2020 Mar 16;15(3):e0229540. doi: 10.1371/journal.pone.0229540 (PMC7075569; doi:10.1371/journal.pone.0229540)
Supplement: S2 Appendix — (PDF) [file pone.0229540.s002.pdf]

S2 Appendix: Presentation discussion points

Genetics and Genomics

- Genetics definition – study of a person's genome or genetic make-up
- Genome (Genomics) definition – the complete set of genetic make-up of an individual or organism
- Genetic Testing Potential Benefits
  - Early detection of disease risk
    - Prevention based on disease risk
  - Tailored treatment – prescriptions, food, allergies, cancer
  - Family planning
  - Better understanding of why some populations and communities get sick and others don't.
- Genetic Testing Potential Risks
  - Emotional response to bad news
  - No treatment for identified conditions
  - Results could be wrong (False Positive, False Negative)
  - Genetic result may reveal genetic information about other family members or community members.
  - Study participants may feel uncomfortable giving biological samples like blood, saliva or stool.
  - Study participants may feel uncomfortable answering detailed questions about their life or family history.
  - If new drugs or health care products are produced, they may be expensive and may not be available to everyone.
  - Although health information is de-identified and well protected, it is not possible to guarantee complete security. Study participants' could be breached.
  - Information about study participants could be misused, like to deny someone a job or health or life insurance
  - This research could find associations between racial or ethnic group characteristics and health problems like addiction or mental illness. Findings like these could be misused to stigmatize or discriminate against people in that group, even if they did not participate in the study.

Research at SCF

- 2005 - SCF Research Policy
  - All research with SCF customer-owners, within SCF facilities, and/or involving SCF employees requires prior approval before study activities can begin
  - Reviewed by research committee
  - Final review with approval/denial by SCF Board of Directors
- 2006 - SCF Research Department established
- Fit with SCF vision, mission, operational principles, and family wellness objectives
- Alaska Native and American Indian people are partners in the research and treated with respect
- Research is on an important topic, of good quality, and *results must be returned to the community*
