# Supplementary material for: An Alaska Native community’s views on genetic research, testing, and return of results: Results from a public deliberation
Source: PLoS One. 2020 Mar 16;15(3):e0229540. doi: 10.1371/journal.pone.0229540 (PMC7075569; doi:10.1371/journal.pone.0229540)
Supplement: S3 Appendix — (PDF) [file pone.0229540.s003.pdf]

S3 Appendix: Opinion poll

Return of Genomic Test Results at Southcentral Foundation (SCF)

1. It is important for SCF to offer genomic testing.
   - □ Strongly Disagree
   - □ Disagree
   - □ Agree
   - □ Strongly Agree
   - □ Unsure

2. Genomic test results would benefit the healthcare I receive at SCF.
   - □ Strongly Disagree
   - □ Disagree
   - □ Agree
   - □ Strongly Agree
   - □ Unsure

3. Results of a genomic test should be returned to a customer-owner even if we don't know what the results mean?
   - □ Strongly Disagree
   - □ Disagree
   - □ Agree
   - □ Strongly Agree
   - □ Unsure

4. I would not need help interpreting my genomic test results.
   - □ Strongly Disagree
   - □ Disagree
   - □ Agree
   - □ Strongly Agree
   - □ Unsure

5. I would want to receive the results of a genomic test, even if it showed I had a risk for an untreatable or incurable health condition.
   - □ Strongly Disagree
   - □ Disagree
   - □ Agree
   - □ Strongly Agree
   - □ Unsure

6. I would share the results of a genomic test with my family member(s) if the results could affect their health.
   - □ Strongly Disagree
   - □ Disagree
   - □ Agree
   - □ Strongly Agree
   - □ Unsure

7. If a genomic test result could have implications for family members, the individual should be required to share their genomic test results.
   - □ Strongly Disagree

- ◻ Disagree
- ◻ Agree
- ◻ Strongly Agree
- ◻ Unsure

8. SCF should provide support for individuals who take a genomic test, like employing a genomic counselor, even if that means taking away funding from another health program at SCF.
   - ◻ Strongly Disagree
   - ◻ Disagree
   - ◻ Agree
   - ◻ Strongly Agree
   - ◻ Unsure

9. It is important for customer-owners to know the results of genomic tests, even if we do not know what the results mean.
   - ◻ Strongly Disagree
   - ◻ Disagree
   - ◻ Agree
   - ◻ Strongly Agree
   - ◻ Unsure

10. Providers should make treatment recommendations based on genomic research, even though research is always being updated.
    - ◻ Strongly Disagree
    - ◻ Disagree
    - ◻ Agree
    - ◻ Strongly Agree
    - ◻ Unsure

11. Providers should be able to choose a medical treatment, even if a genomic test shows the treatment will not work for the customer-owner.
    - ◻ Strongly Disagree
    - ◻ Disagree
    - ◻ Agree
    - ◻ Strongly Agree
    - ◻ Unsure

12. Customer-owners should be able to choose a medical treatment, even if a genomic test shows the treatment will not work for the customer-owner.
    - ◻ Strongly Disagree
    - ◻ Disagree
    - ◻ Agree
    - ◻ Strongly Agree
    - ◻ Unsure

13. Customer-owners should be able to get a genomic test, even if having the test available means other programs would have less funding.
    - ◻ Strongly Disagree
    - ◻ Disagree

- ◻ Agree
- ◻ Strongly Agree
- ◻ Unsure

14. Customer-owners should be able to get a genomic test, even if it might not help.
    - ◻ Strongly Disagree
    - ◻ Disagree
    - ◻ Agree
    - ◻ Strongly Agree
    - ◻ Unsure

15. SCF should limit when providers can order a genomic test.
    - ◻ Strongly Disagree
    - ◻ Disagree
    - ◻ Agree
    - ◻ Strongly Agree
    - ◻ Unsure

16. SCF should share genomic test results with family members of an individual who took a genomic test and results could implicate their family member's health.
    - ◻ Strongly Disagree
    - ◻ Disagree
    - ◻ Agree
    - ◻ Strongly Agree
    - ◻ Unsure

17. A customer-owner has a genomic test done and it shows treatment A would be better for customer-owner than treatment B, should these types of genomic results be returned to the customer-owner?
    - ◻ Strongly Disagree
    - ◻ Disagree
    - ◻ Agree
    - ◻ Strongly Agree
    - ◻ Unsure

18. Resources should be used for purposes other than genomic testing.
    - ◻ Strongly Disagree
    - ◻ Disagree
    - ◻ Agree
    - ◻ Strongly Agree
    - ◻ Unsure
