# Supplementary material for: An Alaska Native community’s views on genetic research, testing, and return of results: Results from a public deliberation
Source: PLoS One. 2020 Mar 16;15(3):e0229540. doi: 10.1371/journal.pone.0229540 (PMC7075569; doi:10.1371/journal.pone.0229540)
Supplement: S4 Appendix — (PDF) [file pone.0229540.s004.pdf]

S4 Appendix. Scenarios

#1 – Research
Recently while visiting your provider at your health clinic, you were invited to participate in a genetics study being conducted with/in your community to help understand the high rates of heart disease your people are said to suffer from. You have never participated in a research study before, but heart disease runs in your family and you think this research may be beneficial. Some scientists believe that the key to solving the high rates of disease in specific populations is in understanding and documenting the genes of those individuals. However, your friend, who is also a member of the community, has publicly raised questions about the need to study genetics for a disease with known links to diet and lifestyle behaviors. The local research review board approved the genetics project as a worthwhile study in spite of these concerns, and concluded that the study presents little risk, if any, to the participants. You are interested in participating in this study that was deemed to be worthwhile, but you also remember hearing stories about genetics projects in the past that targeted Native communities did not always benefit the Native community. Discuss what you think about genetics research in your community.

#2 – Research
A genetics study led by a young scientist who is also Alaska Native is beginning to be discussed in your community. The project would take saliva samples from the entire community to analyze the microbiome of all community members. The microbiome is the collection of microbes, or bacteria, that live in and on our bodies. The researcher tells you that saliva is a great specimen to work with because it is rich with bacteria while still containing a small amount of your DNA, as well. Even though your DNA can be identified within your saliva sample, the researcher tells you they are only analyzing the microbial DNA and not the human DNA content. The project promises to be painless and very informative to the scientific community which may eventually lead to information that could have beneficial health implications for everyone. You wonder if sampling the entire tribe is necessary and where the DNA and data ends up upon the completion of the project. The community advisory board has split opinions on whether to approve the project. Some members think the project is innovative and represents progress for your community. Other community advisory members worry about the security of tribal genomic resources and data. Discuss the issues and your opinions.

#3 – Genetics Testing
Many people know that when you have a child a blood spot test (a “heel stick” test) is done. The test will determine if the baby has a number of disorders that could be treated to prevent developmental struggles and future disability. Imagine, though, that you have just had a baby and rather than a blood spot test that looks for specific disorders, your provider recommends a “genome-wide” test that might find many other possible illnesses. Unlike the heel stick test, a genome-wide test explores a person’s entire collection of genes, not just for specific illnesses. This test will still only require a small drop of blood. However, it will return many more results that may tell you about your child’s future health, including risks for heart disease or breast cancer. The doctor explains that in the unlikely event your child has a risk for a certain disease, breast cancer for example, that possibly a healthy lifestyle will prevent it from occurring. The consent form informs you that your baby’s data will become part of a collective set of data, as well as, your child’s health record. This information raises a number of questions. Who has access to your child’s medical record, and will the information be kept private? Does a result

showing your child to be a genetic risk for some disease mean that you too are at risk for this disease? And, what other information could possibly be taken from these results?
In your small group, discuss the most pressing reasons why or why not to choose either test (blood spot vs genome-wide). What sort of benefits or risks are associated with the blood spot test versus the genomic wide test?

#4 – Genetics Testing
Breast cancer is a serious concern for both women and men, and it is reported that about 10% of breast cancers are inherited. Certain genetic mutations can indicate you may be at risk for developing breast cancer. Imagine that your local provider is part of a pilot program to provide genetics tests to your entire community in order to detect early and treat illnesses, like breast cancer. As a research project, the pilot program will analyze the results and make inferences about your community's health. The test shows that you have mutations that may put you at risk for developing breast cancer. In fact, many of your community report receiving this same result. Now, you find that many of you are having further doctor's, dieticians, and physical training appointments that are becoming time consuming and costly. In your various appointments you learn that you need to live a healthy lifestyle and eat nutritiously. Do you think that all communities would benefit from this type of testing and healthcare? Are their any risks associated with this situation? In your small groups, discuss your opinions.

#5 – DTC Genetics
After participating in a direct-to-consumer genetics tests, like 23andme or AncestryDNA, you find that you have a marker for Celiac disease. Celiac disease is the illness that keeps people from being able to digest gluten and is a genetically inherited, but no one in your family is gluten intolerant; neither of your parents or children have this illness. You take this report to your provider to help you understand your new illness. Your doctor explains the sickness to you and assures you that the integrated dieticians at your clinic will be happy to help if you have the illness. However, the doctor wants to conduct her own tests to confirm this direct-to-consumer result. You believe it would be best if your provider did the genetics test on your entire family, but the doctor refuses. Your provider informs you that genetic screenings are expensive, and that the clinic only orders them when it is necessary. After several invasive tests you are confirmed to have Celiac disease. After changing your diet at the recommendation of the dietician you overall sense of well-being seems improved, which if interesting, because you didn't know you were sick. What are the benefits and risks of providing full genetics screening for all customer-owners to perhaps improve overall community health?

#6 – DTC Genetics
Direct-to-consumer genetics tests, like 23andme or AncestryDNA, are a popular way to explore your genetics for a relatively small fee at home. These tests will give you a report on where they believe your ancestors are from as well as information on your health risks. You decide to do one of these tests and are surprised by your health results! The test says you are at risk for Alzheimer's disease. You are devasted and immediately take this report to your doctor. Your doctor attempts to calm you by explaining the result, but he only confuses you. Now you aren't sure who to believe and wonder if you need a second opinion. The second opinion doctor states that your risk is very low and that many people probably carry the same low risk as you. This doctor also tells you that in the unlikely event you do show symptoms someday there are early

treatments available. Can you start the early treatment now to be safe? Will your family provider alert your immediate family of your result for the implications it could have on their health? What other concerns do you have about this result or your future health? Discuss these concerns and possibilities.
