# Supplementary material for: An Alaska Native community’s views on genetic research, testing, and return of results: Results from a public deliberation
Source: PLoS One. 2020 Mar 16;15(3):e0229540. doi: 10.1371/journal.pone.0229540 (PMC7075569; doi:10.1371/journal.pone.0229540)
Supplement: S5 Appendix — (PDF) [file pone.0229540.s005.pdf]

S5 Appendix. Final summary report

Deliberation on Genomic Results at Southcentral Foundation

Southcentral Foundation (SCF) Research Department convened a public deliberation on January 25 – 26, 2019 to talk about genetics and genomics research, testing, and return of results at SCF. Nineteen customer-owners participated. Participants ranged in age from 22 – 63 years. Some of the participants were also employees of SCF.

The deliberation began with a presentation about genetics and genomics research and testing and the conduct of research at SCF. Participants discussed three issues: (1) potential benefits; (2) potential risks; and (3) questions about governing policies.

Many participants emphasized the potential for genetics and genomics research and testing to:

1. Empower and improve the health of individuals and the community and
2. Expose individuals and communities to privacy breaches of personal information, discrimination, and emotional harms.

Throughout the deliberation, participants underscored the benefit/risk tradeoffs of genetics and genomics.

Benefits of genetic testing	Risks of genetic testing
• capable of promoting family connections	• potentially exposing paternity information in harmful ways
• an empowering means to participate in your own preventative health care	• questioned regulations including for-profit companies and credibility of these tests
• importance of engaging family members in discussions about genetics as part of their overall approach to health care	• concerns that including families in personal health care decisions could challenge individual autonomy and privacy
• promotion of individual autonomy, privacy and empowerment to make informed decisions	• collective benefits or value of genetic and genomic research results might also diminish an individual’s autonomy
• potential for genetic research to empower individuals in making their own health care decisions	• decisions about genetic research should be made against the backdrop of a long history of collective harms and injustices

The impacts of genetics research discussion highlighted important distinctions between individual and collective concerns. Collective harms that included: shared histories of trauma, communal harm associated with loss of traditional dietary practices, and damage to shared environments. Many related to stories shared of collective harm, which served as a basis for understanding the potential risks involved in genetics research and the potential for genetic research to perpetuate collective breaches of mistrust and violation of communal values.

Finally, participants carefully considered aspects of SCF that would need to be addressed if genetic and genomic research and testing were integrated into regular health care delivery. All participants returned for both days. Many participants remained actively engaged throughout the entire deliberation. All individuals participated at some point during the event. Many individuals offered concluding remarks that expressed satisfaction with the event. Participants expressed interest in additional opportunities to participate in research with SCF.
